# Optimizing the HRP-2 *in vitro* malaria drug susceptibility assay using a reference clone to improve comparisons of *Plasmodium falciparum* field isolates

**DOI:** 10.1186/1475-2875-11-325

**Published:** 2012-09-13

**Authors:** Wiriya Rutvisuttinunt, Suwanna Chaorattanakawee, Stuart D Tyner, Paktiya Teja-isavadharm, Youry Se, Kritsanai Yingyuen, Panjaporn Chaichana, Delia Bethell, Douglas S Walsh, Chanthap Lon, Mark Fukuda, Duong Socheat, Harald Noedl, Kurt Schaecher, David L Saunders

**Affiliations:** 1 Department of Immunology and Medicine, US Army Medical Corps, Armed Forces Research Institute of Medical Sciences (USAMC-AFRIMS), Bangkok, Thailand; 2 Armed Forces Health Surveillance Center, Silver Spring, MD, USA; 3 The National Center for Parasitology, Entomology and Malaria Control (CNM), Ministry of Health, Phnom Penh, Cambodia; 4 Medical University of Vienna, Vienna, Austria

**Keywords:** Malaria, *Plasmodium falciparum*, HRP-2, ELISA, Anti-malarial drugs, Drug resistance, Drug susceptibility test

## Abstract

**Background:**

Apparent emerging artemisinin-resistant *Plasmodium falciparum* malaria in Southeast Asia requires development of practical tools to monitor for resistant parasites. Although *in vitro* anti-malarial susceptibility tests are widely used, uncertainties remain regarding interpretation of *P. falciparum* field isolate values.

**Methods:**

Performance parameters of the W2 *P. falciparum* clone (considered artemisinin “sensitive”) were evaluated as a reference for the HRP-2 immediate *ex vivo* assay. Variability in W2 IC_50_s was assessed, including intra- and inter-assay variability among and between technicians in multiple experiments, over five freeze-thaw cycles, over five months of continuous culture, and before and after transport of drug-coated plates to remote field sites. Nominal drug plate concentrations of artesunate (AS) and dihydroartemisinin (DHA) were verified by LC-MS analysis. *Plasmodium falciparum* field isolate IC_50_s for DHA from subjects in an artemisinin-resistant area in Cambodia were compared with W2 susceptibility.

**Results:**

Plate drug concentrations and day-to-day technical assay performance among technicians were important sources of variability for W2 IC_50_s within and between assays. Freeze-thaw cycles, long-term continuous culture, and transport to and from remote sites had less influence. Despite variability in W2 susceptibility, the median IC_50_s for DHA for Cambodian field isolates were higher (p <0.0001) than the W2 clone (3.9 nM), both for subjects with expected (less than 72 hours; 6.3 nM) and prolonged (greater or equal to 72 hours; 9.6 nM) parasite clearance times during treatment with artesunate monotherapy.

**Conclusion:**

The W2 reference clone improved the interpretability of field isolate susceptibility from the immediate *ex vivo* HRP-2 assay from areas of artemisinin resistance. Methods to increase the reproducibility of plate coating may improve overall assay interpretability and utility.

## Background

Artemisinins are the latest class of safe, effective anti-malarial drugs to be threatened by drug resistance [1,2]. Assessment of artemisinin therapeutic efficacy is essential for health policy decision making for malaria control programmes, but an operational definition of artemisinin resistance remains elusive. *In vitro/ex vivo* parasite drug susceptibility tests are widely used, but uncertainties remain regarding the use and interpretation of such systems to evaluate field isolates for artemisinin resistance, particularly when used in isolation, without clinical correlation. The most commonly used *in vitro/ex vivo* susceptibility assays, including histidine-rich protein 2 enzyme linked immunosorbent assay (HRP-2 ELISA) [3,4], schizont maturation [3,5], radioisotopic uptake [6,7], and most recently the SYBR Green I method [8-11], are subject to numerous sources of variability. Differences in assay methods, laboratory conditions, parasite strain susceptibility phenotypes, and technical performance can confound interpretation and affect comparability of results [12-14], especially across different laboratories. In addition, drug properties, including solubility and mechanism of action can have method-dependent effects on data analysis and interpretation [15].

The need for harmonized *in vitro/ex vivo* drug susceptibility monitoring to be employed by a network of regional reference laboratories has been well articulated [13]. A key recommendation supporting this effort is the use of standardized *Plasmodium falciparum* reference clones to provide context for variability among the several assay systems. Quantifying inherent assay variability and establishing a drug susceptibility reference range are essential for laboratory-adapted clones to permit meaningful data comparisons. Just as in clinical laboratories, establishing reference ranges can account for local variance attributable to technical performance and methodology issues. Reference ranges can then be used to classify susceptibilities of *P. falciparum* field isolates obtained from different laboratories.

Since 2004, AFRIMS has used an HRP-2 ELISA method for drug resistance testing because of its high sensitivity, particularly for the artemisinins, when used in immediate *ex vivo* assays of *P. falciparum* field isolates from low-transmission endemic areas, such as Thailand and Cambodia [3,16]. AFRIMS selected the “Indochina” W2 clone as a reference for its genetic similarity to potential field isolates, its drug susceptibility profile, and ease of culture adaptation.

W2 drug susceptibility was measured by 50% inhibitory concentrations (IC_50_) against dihydroartemisinin (DHA) and artesunate (AS), as well as key long-acting agents, including mefloquine (MQ) and chloroquine (CQ), which were used as drug susceptible and resistant controls respectively. Experiments were conducted to systematically assess potential sources of assay variability. Variations in IC_50_s were quantified, and the susceptible ranges of W2 for DHA and AS were established in the HRP-2 assay. The susceptibility range for W2 clones used as quality controls was also compared against *P. falciparum* field isolates from an area of suspected artemisinin resistance in western Cambodia [17].

## Methods

### *Plasmodium falciparum* W2 clone cultivation

*Plasmodium falciparum* reference clone W2, maintained in culture continuously at AFRIMS and cryopreserved in a glycerolyte solution, was recovered as previously described [18]. Continuous cultures were grown using a modified Trager and Jensen method [19]. Briefly, parasites were maintained in RPMI-1640 medium containing 25 mM HEPES, 25 mM sodium bicarbonate, 5% human O^+^ erythrocytes, 10% pooled AB^+^ serum and 0.1 mg/mL gentamycin at 37°C with 5% CO_2_, 5% O_2_, and 90% N_2_. Human erythrocyte and pooled serum were obtained from the National Blood Center, Thai Red Cross Society. Parasites were synchronized utilizing 5% D-sorbitol as previously described [20]. Two sorbitol treatments were performed with a 64-hour interval to provide the highest proportion of synchronized parasites, and parasite culture was maintained for 48 hours after synchronization prior to conducting the drug susceptibility assay.

### Preparation of drug-coated plates

Anti-malarial drugs were coated onto 96-well plates in triplicate to assess W2 clone variability, with the exception of the drug stability and field isolate experiments where duplicate wells were used. Drug compounds were dissolved in 70% ethanol to make 1 mg/ml stock solutions, and then diluted to appropriate concentration by sterile distilled water. Three-fold serial drug dilutions were performed on plates which then air-dried overnight in a biosafety cabinet to reach final nominal drug concentrations (after 200 μL of sample were added) ranging from 20 to 0.027 ng/ml for DHA and AS, 200 to 0.274 ng/ml for MQ and 2,000 to 2.74 ng/ml for CQ. The top row of each plate served as a drug-free control. Molecular weight of DHA, AS, MQ (HCl) and CQ (2H_3_PO_4_) were 284.35, 384.42, 414.78, and 515.92 g/mole, respectively. Dried plates were stored at 4°C. Plates were used within 2 months of preparation. Plate preparation and storage methodology was based on preliminary experiments establishing reproducibility of IC_50_s under these conditions.

### Analysis of plate drug concentration by LC/MS

Drug substance from the starting wells of three lots of three dry drug-coated plates selected for quality control analysis was recovered to determine accuracy of the nominal starting concentration of 4 ng of AS or DHA in each well. The solvent for AS and DHA used for LC/MS analysis (100 μL of 50% acetonitrile) was added to each starting well to obtain a final concentration 40 ng/ml for both AS and DHA. The coated drug was dissolved in the solution by manually agitating the plate for 5 min with complete dissolution. The resulting solution was then transferred to an auto-sampler vial for LC/MS analysis, and 5 μL of the solution was injected directly into the system for quantification.

Plate drug concentrations for AS and DHA for different coated plates lots were verified by LC/MS analysis using a previously described bio-analytical method [21]. Briefly, AS and DHA were analysed using the LC/MS system consisting of a reversed phase column (XTerra MS C18, 3.5 μm, 2.1 x 50 mm, Waters Corporation, MA, USA) and pre-column of the same material (2.1 x 10 mm) connected to the Alliance 2695 Liquid Chromatography system equipped with a single quadrupole Micromass ZQ (MM1) Mass Spectrometry detector (Waters Corporation) in the positive electrospray ionization mode and single ion recording. The analytes (AS and DHA) were eluted from the column with 6.25 mM ammonium acetate buffer pH 4.5 and acetonitrile gradient from 20% to 40% in 9 min at the flow rate of 0.4 ml/min. The ammonium-adducts of DHA and AS were detected at the m/z ratios of 302 and 402 and eluted near 5 and 6 min, respectively, with total analysis time of 12 min. Resulting concentrations were extrapolated from a standard curve of DHA or AS respectively.

### HRP-2 ELISA drug susceptibility testing

Synchronized W2 parasites with >90% ring forms were diluted to 0.5% parasitaemia with 1.5% haematocrit in 0.5% Albumax RPMI-1640, and transferred to drug-coated plates. Plates were incubated at 37°C with 5% CO_2_, 5% O_2_, and 90% N_2_ for 72 hours. For field isolates, blood samples with ≤0.5% parasitaemia were transferred to drug-coated plates without culture adaptation (*ex vivo*), and those with >0.5% parasitaemia were diluted to 0.2-0.5% before transferring. Plates were incubated at 37°C using a candle jar, instead of mixed gas, to create favourable conditions, and parasite growth inhibition after 72 hours was analysed by HRP-2 ELISA assay, as previously described [3].

Due to the high sensitivity of the HRP-2 ELISA assay, in order to obtain IC_50_ values, samples were diluted before performing the ELISA. To estimate parasite growth rate, samples were collected from the untreated wells at 0, 24, 48 and 72 hours, then serially diluted and analysed by HRP-2 ELISA assay. The serial dilution providing the greatest increase in OD values between 0 and 72 hours was then used as the dilution factor for the parasite culture samples in the HRP-2 ELISA. HRP-2 protein ODs were plotted against drug concentrations and IC_50_s were analysed using ICEstimator 1.2 by non-linear regression, to estimate the sigmoidal dose response curve as previously described [22,23]. For the purposes of analysing sources of variability in the W2 clone experiment (with the exception of the drug stability experiment), three individual IC_50_ values were estimated from each of the triplicate serial drug dilutions on the plate, while IC_50_ values from field isolates and the drug stability experiment were calculated by averaging ODs from duplicate serial dilutions.

### Assessment of sources of assay variability

*In vitro* drug susceptibility testing of the W2 parasite was conducted systematically in order to determine variability of the following factors: (1) variability within an HRP-2 assay found between wells of drug plates and between different drug plates run by a single technician on a single day (‘Intra-assay’ variability); (2) variability between assays conducted on five different days by the same technician (‘Inter-assay’); (3) Technician performance assessed as the variability between technicians in overall median IC_50_ values; (4) variability introduced by repeated parasite freeze/thaw cycles; (5) variability due to long-term parasite culture (Longevity); and, (6) Stability of drug-coated plates during transport to/from and storage at field sites, assessed as difference in IC_50_s analysed on drug-coated plates before and after transport to field sites. During transport to and from the field sites, plates were stored on ice packs (2-6°C) en route, with an average of eight hours transport time, and stored in a refrigerator (2-6°C) at the field site.

### Comparative drug susceptibility of W2, 3D7 and D6 clones

In order to establish comparability with previous published reports of parasite clone susceptibility phenotypes, *in vitro* susceptibility results for W2, 3D7 and D6 against DHA, AS, MQ and CQ (eight replicates each) were compared.

### Artemisinin susceptibility of field *Plasmodium falciparum* isolates against reference W2 clone

Clinical isolates were obtained from patients enrolling in a previously reported clinical trial of artesunate monotherapy in Tasanh, Cambodia [17]. *Immediate ex vivo* (IEV) drug susceptibility testing without culture adaptation was performed on 125 evaluable *P. falciparum* isolates from an area of known artemisinin resistance using the methods above. Isolates were considered evaluable if an IC_50_ could be determined using the IEV method. In each 12-plate coating lot used for field parasite analysis, two to seven drug plates were randomly selected for W2 drug susceptibility testing either before or after transport to field. IEV IC_50_ values of field isolates were compared with W2 IC_50_ values.

### Statistical analysis

Geometric mean, median, range and interquartile values, and% CV for IC_50_ values were calculated. To define the normal susceptible performance range of W2 for DHA and AS, outlier IC_50_ values, as determined by Grubbs' test, were excluded and the range of minimum and maximum IC_50_ values were defined. Kruskal-Wallis and Mann–Whitney U tests were used to analyse the differences in technician performance and compare the IC_50_ values of the W2 clone to field parasite isolates. To assess drug stability, the Wilcoxon matched-pair test was used to compare W2 IC_50_ values on drug-coated plates before and after transport to field sites. Statistical significance was defined as *p <0.05*. For multiple comparison analysis, the cut-off *p-value* was adjusted for the number of comparisons by using the Bonferroni correction. Statistical analysis was performed using Graphpad Prism for Windows, version 5 (Graphpad Software, Inc, San Diego, CA, USA).

## Results

### Assessment of sources of assay variability

**Figure 1:**
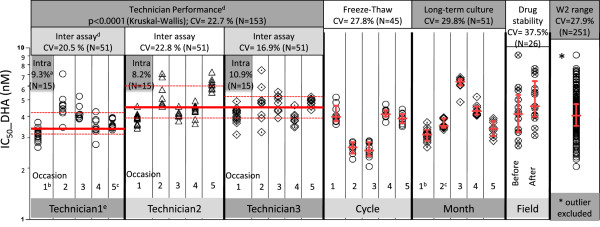
** HRP-2 ELISA assay variability for the W2 *Plasmodium falciparum *parasite clone.** Each data point represents an individual IC_50_ value obtained from triplicate columns on each of three or five drug-coated plates, generating nine or 15 IC_50_ values per experiment/occasion. For the drug stability experiment under field conditions, ODs from duplicate columns were averaged to generate a single IC_50_ value for each plate, producing 26 IC_50_ values for 26 assays run. All individual IC_50_ values are presented in the “W2 Range” column. Red lines represent median IC_50_s and interquartile ranges. ^(a)^ ‘Intra-assay’ variability is assessed by% CV of IC_50_ values within each HRP-2 assay conducted by a single technician on a single day. ^(b), (c)^ IC_50_ values measured from the same experiment were analysed in both the intra/inter-assay variability and long-term culture variability. ^(d)^ ‘Inter-assay’ variability was assessed by the % CV of IC_50_ values among HRP-2 assays conducted on different days by the same technician, while technician performance was assessed by the difference in IC_50_ values among technicians. ^(e)^ IC_50_ values obtained from technician 1 were significantly lower than those from technician 2 and 3.

Three technicians performed IC_50_ assays on the W2 clone concurrently using triplicate serial dilutions on five different plates for each drug assayed to obtain 15 individual IC_50_ values (Figure 1). The% CV for “intra-assay” variability from these individual experiments was consistent between technicians, ranging from 8.2%-10.9% for DHA (Figure 1) and 8.0%-15.9% for AS.

Each technician repeated this experiment on a total of five occasions to assess the day-to-day variability of IC_50_ values (inter-assay variability). The% CV values for inter-assay variability ranged from 16.9% to 22.8% for DHA (Figure 1) and 32.3% to 36.1% for AS. Median IC_50_ values for the three technicians ranged from 3.6 to 4.3 nM with an overall% CV of 22.7 for DHA (Figure 1) and 3.6 to 5.4 nM (% CV = 36.2 overall) for AS. There was a statistically significant difference in IC_50_ values obtained from each of the three technicians for both DHA and AS (*p < 0.0001* by Kruskal-Wallis test). A significant difference in median IC_50_ values was found between technician 1 and technician 2, as well as technician 1 and 3 (*p < 0.0001*, Mann–Whitney U tests), but not between technician 2 and 3 (*p = 0.84*).

IC_50_ assays were conducted following parasite freeze-thaw for up to five cycles (Figure 1). Median IC_50_ values in five separate freeze/thaw experiments were 2.5-4.1 nM for DHA (% CV = 27.8) and 2.7-3.9 nM for AS (% CV = 22). To assess variability due to long-term parasite culture, IC_50_ values were evaluated monthly over a five-month period using a continuously growing W2 culture (Figure 1). During this time, median IC_50_ values fluctuated between 3.0 and 6.4 nM for DHA (% CV = 29.8) and 3.0 and 6.7 nM for AS (% CV = 37.6).

The effects of drug-coated plate stability during transport to/from and storage at the field on W2 IC_50_ were evaluated, with 26 paired IC_50_ assays performed on 13 different drug-coated plate lots prior to transport, and upon return from remote field sites after several weeks (maximum two months) of storage on-site. Despite IC_50_ variability (% CV) between individual plate lots for W2 with both DHA (% CV = 37.5) and AS (% CV = 49.2), there were no statistically significant differences in IC_50_ values obtained from plates before going and after returning from the field for either DHA (mean difference for DHA −0.64 nM for pairwise comparisons using the Wilcoxon matched-pair test; p = 0.06) or AS (difference of 0.08 nM; *p = 0.74*, see Figure 1).

When a total of 251 W2 IC_50_ values generated from all experiments were pooled, and outliers were excluded using Grubb’s test, median IC_50_ of DHA was 4.1 nM (IQR 3.5-4.7 nM), and AS was 4.2 nM (IQR 3.4-6.0 nM). The overall IC_50_ range was 2.0-7.6 nM for DHA, and 2.0-9.4 nM for AS. Using the same plate lots, the median IC_50_ of CQ for the W2 clone was 297.4 nM (IQR 221.3-433.7 nM) and 21.3 nM for MQ (IQR 18.3-31.7 nM) (Table 1). The W2 clone was resistant to CQ but susceptible to mefloquine when compared to other commonly used clones D6 and 3D7, while 3D7 was less artemisinin-sensitive than W2 and D6 (Table 2).

**Table 1:** **Overall variation in IC**_**50**_**values (nM) of DHA, AS, MQ and CQ against the W2 clone measured from all validation experiments (Outlier IC**_**50**_**values were excluded)**

**Drug**	**N**	**Geometric mean**	**Median**	**Maximum**	**Minimum**	**IQR**
DHA	250	4.1	4.1	7.6	2.0	3.5-4.7
AS	250	4.4	4.2	9.4	2.0	3.4-6.0
MQ	248	17.7	21.3	78.1	3.2	18.3-31.7
CQ	251	304.5	297.4	779.1	116.3	221.3-433.7

**Table 2:** **Median IC**_**50**_**s (with interquartile ranges) of DHA, AS, MQ and CQ against W2, D6 and 3D7 clones in the HRP-2 assay (N = 8 each)**

**IC**_**50**_	**DHA (nM)**	**AS (nM)**	**MQ (nM)**	**CQ (nM)**
W2	3.8 (2.6-3.9)	4.2 (3.2-7.1)	30.4 (11.6-46.1)	336.7 (310.3-405.1)
D6	3.9 (3.8-4.3)	5.5 (3.38-7.71)	82.7 (68.2-93.0)	16.1 (6.0-18.3)
3D7	6.0 (4.6-7.3)	10.2 (5.2-11.4)	66.3 (60.8-76.1)	21.9 (10.2-28.3)

**Figure 2:**
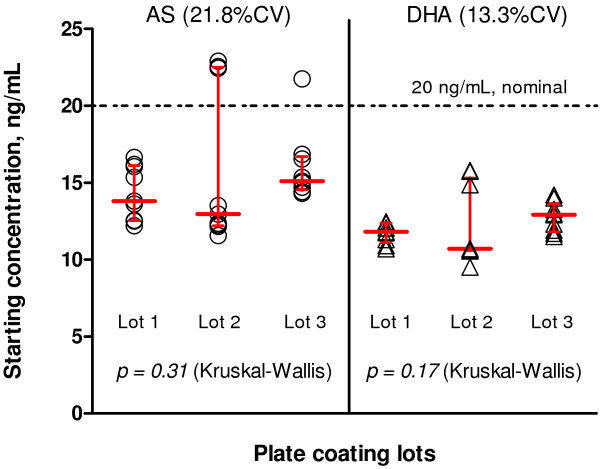
**Starting AS and DHA concentrations used for serial dilutions on *in vitro *drug culture plate lots for the HRP-2 assay.** The dashed line indicates the nominal final starting concentration of 20 ng/mL after addition of sample for both drugs. Red lines indicate medians and interquartile ranges for drug concentration. From three drug-coated plate lots, a total of 27 starting wells collected were measured (three wells/plate x three plates/lot).

Quality control of drug-coated plates was evaluated by LC/MS of starting AS and DHA concentrations, measuring the highest drug concentration in the starting wells used to initiate serial dilutions. Variability was assessed over a period of six months using three different lots of drug-coated plates. Three plates per lot were randomly selected and a total of 27 starting drug concentrations (triplicate wells/plate) were measured (Figure 2). There was variation in starting drug concentration between plates for AS with 21.8% CV, and an overall median starting concentration of 14.7 ng/mL compared to a nominal target starting concentration of 20 ng/mL after addition of sample. For DHA, the median starting concentration was 11.9 ng/mL compared to a nominal value of 20 ng/mL with a 13.3% CV (n = 27 for both drugs). Despite the differences between actual and nominal starting concentrations, there were no statistically significant differences in median starting concentrations between plate lots.

### Artemisinin susceptibility of field *Plasmodium falciparum* isolates compared to the W2 clone

**Figure 3:**
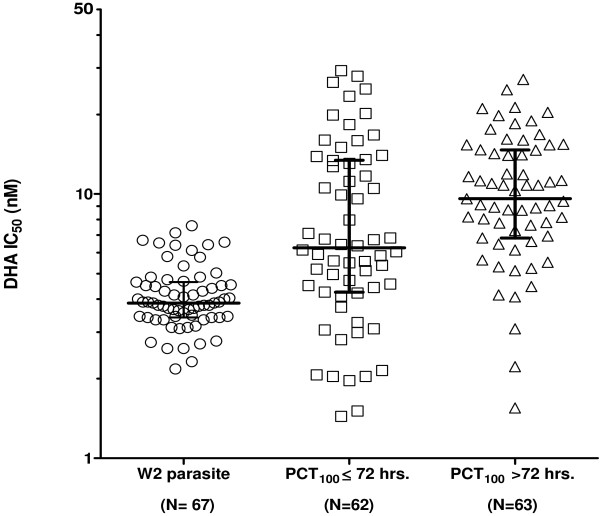
**IC**_**50**_**values of DHA against clinical *Plasmodium falciparum *field isolates collected from a drug-resistant area in Cambodia (middle and right hand columns), compared to the W2 parasite used as a reference clone (left hand column).**Median and interquartile ranges are shown. Median IC_50_s of DHA were significantly higher for patients with PCT_100_ >72 hours (9.6 nM) than PCT_100_ ≤72 hours (6.3 nM, *p = 0.0134*), and both were significantly higher than the reference W2 clone (3.9 nM, *p < 0.0001*).

The IC_50_ values for DHA of field isolates from an artemisinin-resistant area in western Cambodia were compared to the W2 reference clone used for quality control on the same plate lots over a one-year period from August 2008 to August 2009 [17] (Figure 3). There were 12 lots of plates made in total for the study, with IC_50_ values generated for 125 volunteers, and there were no outlier IC_50_ values for the W2 parasite detected or excluded. Despite a broad susceptibility range of the field isolates, there was a significant increase in median IC_50_ for DHA (8.69 nM) compared to the W2 reference clone (3.9 nM) both from patients with 100% parasite clearance time (PCT_100_) ≤72 hours (6.3 nM) and >72 hours (9.6 nM), (*p < 0.0001, Mann–Whitney U test*). Furthermore, the IC_50_ for DHA of field parasite isolates from patients with PCT_100_ >72 hours was significantly higher than PCT_100_ ≤72 hours (*p = 0.0134*). Similarly, median field isolate IC_50_s for AS were significantly higher than those for the W2 clone (*p < 0.0001, Mann–Whitney U test*), and there were significant differences between PCTs greater than or less than 72 hours (6.0 nM vs 5.4 nM, *p = 0.039*).

## Discussion

The value of using an artemisinin-susceptible W2 clone to control for the inherent variability in the HRP-2 *in vitro* assay was demonstrated here, particularly with field isolates, defining a range for drug susceptibility assay performance. By comparing field isolates with a reference clone used for the same plate lots, a clear determination of a reduction in drug susceptibility in an area of reported drug resistance in Cambodia can be attained. This approach has significant public health utility, allowing meaningful comparisons of field isolates populations, and controlling for variability in assay conditions. Although individual or even collective IC_50_ values may offer little in predicting the onset of drug resistance in a community due to wide variability and differences in assay methods and conditions, comparison with a well-characterized clone mitigated some of these limitations on assay interpretation. The use of a reference clone to directly measure reproducibility of IC_50_ values on plates used to assess field isolates may be particularly valuable in accounting for variability due to technique, providing more meaningful inter-laboratory and multi-site surveillance comparisons.

The inherent variability of the HRP-2 ELISA-based IC_50_ assay was quantified and variation in drug concentrations between different plates was found to be an important source of assay variability, confirmed by LC/MS analysis of plate concentrations. While a significant difference in starting drug concentrations between plate-coating lots was not observed, actual drug concentrations recovered from the plates by LC/MS were generally below nominal concentrations, indicating that final IC_50_ may have been overestimated. Greater variation in AS plate concentrations observed compared to DHA may explain somewhat higher variability in AS IC_50_ values compared to DHA. Unlike DHA, the variation seen could be explained by the fact that AS (an esterified butanedioic acid) is readily hydrolyzed to DHA and succinic acid in aqueous solution. Inherent assay variability provides additional impetus to standardize methods wherever possible, and index results to a reference clone. Methods to improve the reproducibility and precision of drug dilutions (e g, use of a pipetting robot, improved stabilizing techniques, or plates with materials that drug substance is unlikely to adhere to) may have the greatest impact on further improving assay reliability. There was significant day-to-day inter-assay IC50 variability for individual technicians (% CV >30 for AS), as well as differences in performance between technicians. Some degree of difference attributable to manual performance of laboratory techniques is expected. Understanding this variability helps to further define the performance range of this assay, and further supports interpretation of field isolate values.

Freeze/thaw cycles, and long-term culture of the W2 clone were less important sources of assay variability, as IC_50_ values generated during culture time/freeze-thaw cycles fluctuated within the assay performance range. Despite an increase in IC_50_ values at month 3 and 4, variability was no greater than that seen between assays performed by the same technician on different days (inter-assay variability), and values at month 5 returned to baseline, suggesting that there were no time-dependent changes in W2 IC_50_ values arising from long-term continuous culture. Transport and storage of drug plates in field conditions did not account for substantial variability, with no difference in W2 IC_50_ values obtained before and after transport to the field, supporting the utility of the HRP-2 method at remote field sites. Unmeasured variability in assay conditions and parasite characteristics may also have contributed. Understanding the inherent variability of the assay, and defining performance ranges of reference parasite clones, are important for laboratories conducting drug susceptibility assays on field isolates.

A reference clone may further be useful in overcoming issues of sampling bias originating from two sources in *in vitro/ex vivo* susceptibility studies. Samples reported from *in vitro/ex vivo* clinical isolate studies are generally derived from clinical trials or dedicated parasite sampling studies. However, in both cases, systematic sampling methods (e g, randomized household cluster surveys) are rarely employed, instead relying on convenience sampling from particular clinics or communities, or retrospective reviews of sample collection efforts from multiple sites and/or laboratories. Recovery of malaria isolates from culture, and interpretability of parasite growth curves are other potential sources of sampling bias, with the proportion of evaluable samples as low as 50-60% in some cases. Low parasitaemia samples are particularly problematic, and the effects on point estimates of ‘resistance’ can be problematic due to the unmeasurable impact of non-recovery on population values. By establishing a susceptibility baseline, comparison with a reference clone mitigates these uncertainties to some extent, facilitating sample comparisons at the population level.

While the W2 clone showed similar trends against W2, D6 and 3D7 in our hands to those previously reported in the HRP-2 assay, the values were not identical, underscoring the need for standardization within and among laboratories. Each *in vitro* assay system has limitations. Because of its high sensitivity, HRP-2 is used as a parasite detection method in several rapid diagnostic test kits [24], and has even been advocated for use in a salivary detection test [25]. HRP-2 is a by-product of parasite metabolism that persists in blood following malaria infection and treatment, making it a poor prognostic indicator. Differences in metabolic conditions during the culture process could potentially confound the interpretation of *in vitro* assay results [26]. The HRP-2 *in vitro* drug sensitivity method presented here has been advocated in populations with low transmission rates, low or fluctuating parasite densities, and chronic infections, and is considered to be acceptable for use in Southeast Asia for these reasons [5,27]. Some have suggested that the SYBR Green I method’s specificity in whole blood may be confounded by the presence of human DNA, limiting its utility in clinical isolates [28]. However, a recent report from Kenya found SYBR Green I to be a sensitive, cost-effective method for field use, and IC_50_ values compared well with historic parasite data using the tritiated hypoxanthine method [12]. Even with the long-accepted “gold standard” hypoxanthine method, wide variability in field isolate values has been reported, most recently in a published review of *in vitro* data collected from drug efficacy trials in Cambodia over seven years. While the large sample size allowed detection of discernible differences in drug susceptibility between specimens collected in the eastern and western portions of the country, such extensive collection efforts may not be feasible in all settings [29].

The reference clone provides a higher level of assurance in the validity and comparability of smaller data sets, and allows for monitoring of trends over shorter periods to support timely public health action, including shifts in drug treatment policy. Comparing changes in field isolate IC_50_s indexed to a well-characterized reference clone that demonstrates within-laboratory reproducibility is recommended. For an assay to have public health utility, the choice of methodology is less important than demonstrated experience with the system, use of a well-characterized reference clone, and most importantly, establishment of reproducible methods.

## Conclusions

The use of standardized *P. falciparum* reference clones to provide context for variability among several assay systems is a key in harmonizing regional laboratories for *in vitro/ex vivo* drug susceptibility monitoring. Quantifying inherent assay variability and establishing a drug susceptibility range of the reference *P. falciparum* clone permitted meaningful data comparisons and improved the interpretability of field isolate susceptibility.

## Competing interests

The authors declare that they have no competing interests.

The views expressed in this article are those of the author(s) and do not reflect the official policy of the Department of the Army, Department of Defense, or the US Government. All human use research received the required ethical approvals from the appropriate authorities.

## Authors’ contributions

Study design: WR, KS, SDT. Conducted experiments, collected and analysed data: WR, SC, SDT, PT, KY, PC, YS, DB, CL, KS. Data interpretation, manuscript preparation: SC, PT, DSW, WR, DLS. Project oversight: SDT, MMF, KS, HN, DS and DLS. All authors read and approved the final manuscript.
